# Thoracoscopic Treatment of Pneumothorax in Marfan Syndrome: Hemostatic Patch to Support Lung Resection Recovery

**DOI:** 10.1155/2018/7597215

**Published:** 2018-09-04

**Authors:** Gloria Pelizzo, Eloisa Arbustini, Noemi Pasqua, Patrizia Morbini, Valeria Calcaterra

**Affiliations:** ^1^Pediatric Surgery Unit, Children's Hospital “G. Di Cristina”, ARNAS Civico-Di Cristina-Benfratelli, Palermo, Italy; ^2^Centre for Inherited Cardiovascular Diseases, Fondazione IRCCS Policlinico San Matteo, Pavia, Italy; ^3^Pediatric Surgery Unit, Department of Maternal and Children's Health, Fondazione IRCCS Policlinico San Matteo, Department of Clinical-Surgical, Diagnostic and Pediatric Sciences, University of Pavia, Pavia, Italy; ^4^Department of Molecular Medicine, Unit of Pathology, University of Pavia, Pavia, Italy; ^5^Pediatric Unit, Department of Internal Medicine, Department of Maternal and Children's Health, Fondazione IRCCS Policlinico San Matteo, University of Pavia, Pavia, Italy

## Abstract

**Introduction:**

In selected patients, the absorbable fibrin patch TachoSil® is superior to standard surgical treatment in reducing air leakage after pulmonary lobectomy. Pulmonary involvement is not considered a main feature of Marfan syndrome (MFS); however, spontaneous pneumothorax (SP) with a high rate of recurrence is frequently reported. We describe the use of TachoSil® in the supportive treatment of recurrent pneumothorax in a girl with MFS.

**Case Report:**

A 12-year-old girl with a previous diagnosis of MFS and recurrent history of left spontaneous pneumothorax was submitted to thoracoscopic atypical lung resection. Two patches (9.5 × 4.8 cm) were cut from the adhesive/foam complex (TachoSil®) and were pressed against the sutured area as supportive treatment. The patient recovered with no further SP recurrences.

**Conclusions:**

The use of the TachoSil® surgical patch may be useful in pneumothorax supportive treatment, particularly in pediatric MFS by ameliorating the mechanical strength of the lung.

## 1. Introduction

Marfan syndrome (MFS) is a connective tissue disorder comprising multiple organ manifestations caused by a mutation in the gene FBN1 that codes for the protein, fibrillin-1 [1]. Although pulmonary symptoms are not generally considered a main feature of Marfan syndrome, many patients have a degree of underlying pulmonary pathology, such as cystic changes, emphysema, spontaneous pneumothorax (SP), focal pneumonia, bronchiectasis, bullae, congenital pulmonary malformations, and apical fibrosis [2, 3]. SP is the most frequent respiratory manifestation in MFS, with an associated prevalence that is between 5 and 11% [3–5]. The optimal SP treatment in MFS remains unclear because of the high rate of recurrence.

TachoSil® is a sterile ready-to-use absorbable patch for intraoperative topical application. In selected patients, TachoSil® is more effective than standard treatment of air leakage after pulmonary lobectomy [6]. Limited data on TachoSil® use in thoracic surgery in the pediatric age are available [7]; thus, we investigated the use of TachoSil® in supportive treatment for recurrent pneumothorax in a girl with MFS.

## 2. Case Report

A 12-year-old girl with a previous diagnosis of MFS was admitted in our Pediatric Surgery Unit with sudden onset of chest pain and shortness of breath after sneezing. Her past medical history revealed two previous left spontaneous pneumothorax (two and three months prior to this hospital admission), managed with conservative measures and pleural drainage.

**Figure 1:**
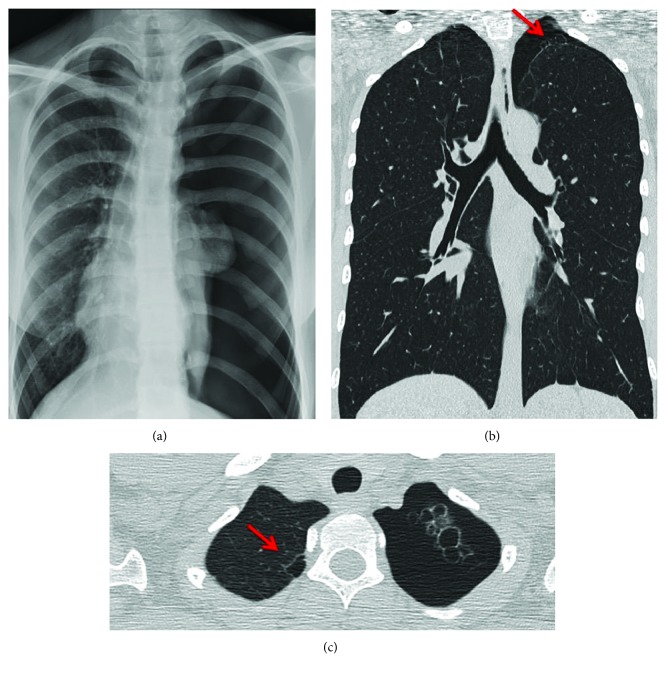
Lung imaging. (a) Chest X-ray. A total collapse of the left lung was showed. (b, c) Thoracic CT. A bullous pulmonary dysplasia was noted (the bullous lesion is indicated with an arrow).

Upon examination, both physical and radiographic findings were compatible with a pneumothorax recurrence on the left side. A thoracic CT scan showed bullous pulmonary dysplasia (Figure 1). Thoracoscopic treatment included resection of the pneumopleural adhesions and bullae. The procedure was performed with the patient in a lateral decubitus position. Three valved ports, ranging in size from 3 to 5 mm, were used. The first port was placed in the mid to the anterior axillary line in the fifth interspace to determine the position of the major fissure and evaluate the lung parenchyma. The position of the fissure dictates the placement of the other ports. The working ports were placed in the anterior axillary line above and below the camera port. Lung resection, including the parenchymal area involved in the lesion, was performed using the LigaSure® device. A lung specimen was removed using a protective specimen endobag through a 5 mm port. No postsurgical drainage tubes were positioned. The patient was discharged on the eighth postoperative day.

**Figure 2:**
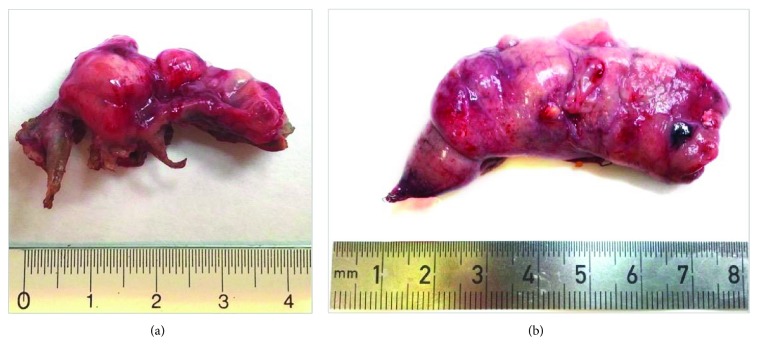
Gross findings of the upper lobe at first (a) and second (b) surgical interventions. Emphysematous changes with marked bulla formation were present.

**Figure 3:**
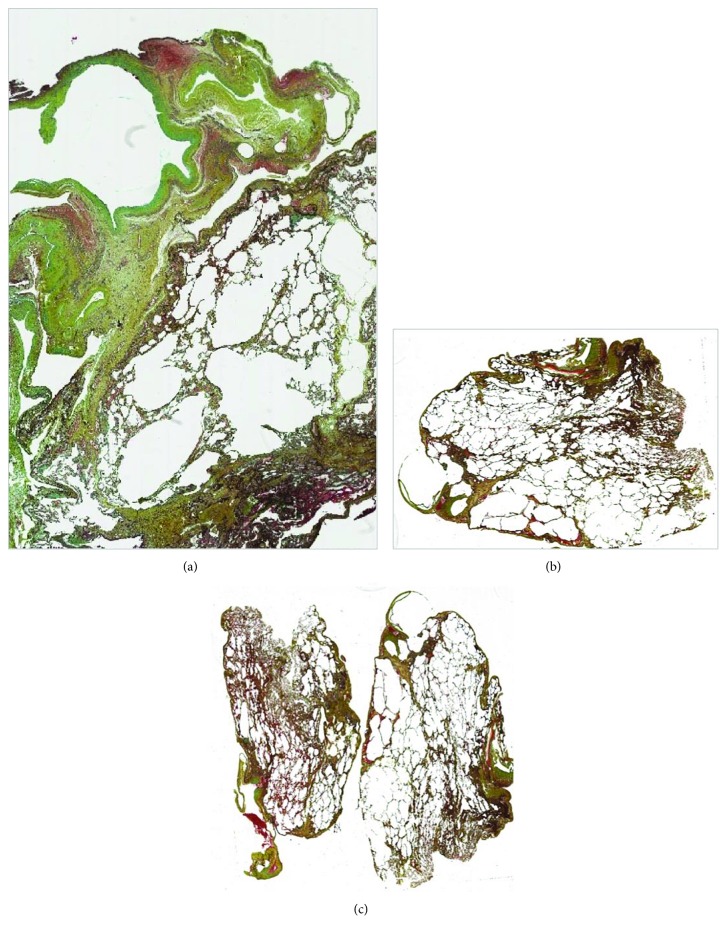
Pulmonary specimen evaluation, after first (a) and second (b, c) surgical interventions; all specimens showed distal acinar emphysema.

One week later, a pneumothorax recurrence necessitated a second thoracoscopic intervention. Further pulmonary resection of the atypical upper left lobe was performed using a stapling device. Two patches (9.5 × 4.8 cm) were cut from the adhesive/foam complex (TachoSil®), rolled and inserted into the 5 mm trocar, and subsequently stretched over the sutures. No drainage tubes were inserted. The postoperative period was uneventful, and the patient was discharged one week later. Gross findings showed severe emphysematous involvement in the upper lobe with marked bulla formation (Figure 2(a)). Microscopic examination revealed a characteristic emphysema morphological pattern juxtaposed to normal lung parenchyma (Figure 3(a)).

Three months later the patient was admitted again, this time with a right-sided pneumothorax. A thoracoscopic intervention on the atypical section of the upper right lobe was performed using a stapling device (Figure 2(b)), and new pieces of the TachoSil patch were applied. No thoracic drainage tubes were inserted. Microscopic findings shown in Figures 3(b) and 3(c) evidenced emphysematous changes. At six-month follow-up, the patient was in good condition with no further pneumothorax recurrences.

## 3. Discussion

MFS is a rare genetic connective tissue disorder with skeletal, ocular, and cardiovascular manifestations [1]. Pulmonary involvement occurs infrequently, with SP being the most commonly reported. However, MFS patients have a PS risk that is 10 times higher than that of the general population [3–5]. The causal gene for MFS, FBN1, encodes for extracellular matrix glycoprotein fibrillin-1, which is found in the lung and is a component of elastic fibres. Abnormalities in fibrillin result in connective tissue friability and laxity [1]. These features, within the lung, can result in flaccidity of small airways and terminal bronchioles, predisposing to premature airway closure, obstruction, and air trapping leading to degenerative changes and emphysema, which may be the main mechanism for pneumothorax in these individuals [8]. Increased mechanical stress in the lung apices of the characteristically tall MFS body habitus is also a consideration [8].

Specific histological changes in a Marfan lung have not been completely characterized in microscopic detail; however, it is commonly accepted that in MFS, the gross pathological condition of the lung corresponds with “fragile lung” at microscopic evaluation [9]. Emphysematous changes have been described in terms of geographic location (e.g., upper vs. lower lobe, bilateral vs. unilateral). In our case, the distal acinar emphysema represents a histologic pattern which correlates with MFS [10] and an underlying history of SP. Dyhdalo and Farver [10] have reported that these changes occur just beneath the pleural surface, while sparing the surrounding acini. Areas of scarring may be present in the distal septa, although with significant separation from other areas of more typical emphysematous changes. Some cases have shown discrete foci of emphysematous changes, while others display more extensive alveolar destruction.

It is however essential to remember that distal acinar emphysema is not a distinguishing feature of MFS and may be seen in other disease processes, notably in tall, thin males with SP. It is important to interpret pathological findings in the context of clinical history and imaging studies.

The relationship between upper lobe pulmonary fibrosis and MFS has also been reported in MFS [11]. Thus, it is tempting to speculate that the fibrosis might be the result of prior scarring caused by stress in the apical sections of the lungs of these tall people, the site where mechanical damage would be expected to be maximal [11].

Therapeutic options for SP include bed rest, oxygen supplementation, manual aspiration, chest tube drainage, and thoracoscopic and surgical interventions [12]. There is a lack of consensus between different international guidelines regarding many aspects of pneumothorax management in the pediatric age. In patients with MFS, treatment should be more aggressive and definitive surgical treatment should be performed at the first occurrence of pneumothorax because of the high rate of recurrence after chest tube treatment. Open surgery and thoracic drainage are not recommended to decrease the risk of the pneumopleural adhesion formation. Thoracoscopic surgery, as a minimally invasive approach, should be considered the treatment of choice. Considering the tissue fragility, stapling devices should minimize trauma and support the suture to prevent air leakage. The TachoSil® surgical patch also favours blood clotting, creates sealing barriers, and facilitates the bonding of tissue components, including pulmonary tissue.

TachoSil® is a sterile ready-to-use absorbable patch for intraoperative topical application. It consists of an equine collagen patch coated with fibrin glue components: human fibrinogen and human thrombin. It thus combines the benefits of a pliable material with the hemostatic and adhesive properties of coagulation factors. The product is currently approved in Europe for supportive hemostatic surgical treatment where standard techniques are insufficient. The efficacy and safety of TachoSil® have been demonstrated in adults following digestive and hepatobiliary surgery (liver resection), in pulmonary lobectomy, in sealing the percutaneous nephrolithotomy tract, kidney resection, open repair of abdominal aortic aneurysm, during splenectomy for oncohemathological conditions, after distal pancreatectomy, for obstetric and gynecologic surgery, inguinofemoral and axillary lymphadenectomy, and in a case of corneal perforation [13–21].

There are few studies which have evaluated the use of TachoSil in pediatric surgery. Vida et al. [22] and Giordano et al. [23] described TachoSil use in children with congenital heart disease who underwent heart surgery in order to control bleeding. Mele et al. [24] reported a preliminary pediatric experience using this hemostatic agent during nephron sparing surgery. Mirza et al. [25] and Lacanna et al. illustrated the use of TachoSil for hemostasis in children undergoing liver transplantation. Molnar et al. [7] described a parenchyma sparing method using a sealant-hemostatic complex foam in a boy suffering from cystic fibrosis of the lung and in a girl with bronchopneumonia in artificial ventilation for acute respiratory distress syndrome and sepsis.

There are no reports in the literature on TachoSil use in pediatric thoracic surgery in MFS. We hypothesized that additional sealing of the anastomosis with a fibrin patch, containing human fibrinogen and thrombin, would improve pulmonary mechanical strength. TachoSil® may be considered effective support in surgical treatment to prevent SP recurrence in children and adolescents with MFS.

## 4. Conclusion

In MFS patients, definitive surgical treatment should be performed at the first occurrence of pneumothorax because of the high rate of recurrence. The use of the TachoSil® surgical patch should be useful in pneumothorax supportive treatment in MFS in order to ameliorate the mechanical strength of the lung, limit the use of postsurgical drainage tubes, and prevent PS recurrence in the pediatric age.
